# The RNA methyltransferase METTL16 enhances cholangiocarcinoma growth through PRDM15-mediated FGFR4 expression

**DOI:** 10.1186/s13046-023-02844-5

**Published:** 2023-10-11

**Authors:** Nianli Liu, Jinqiang Zhang, Weina Chen, Wenbo Ma, Tong Wu

**Affiliations:** Department of Pathology and Laboratory Medicine, Tulane University, 1430 Tulane Avenue, SL-79, New Orleans, LA 70112 USA

**Keywords:** Cholangiocarcinoma, METTL16, PRDM15, FGFR4, m6A modification

## Abstract

**Background:**

RNA N6-Methyladenosine (m6A) modification is implicated in the progression of human cancers including cholangiocarcinoma (CCA). METTL16 is recently identified as a new RNA methyltransferase responsible for m6A modification, although the role of METTL16 in CCA has not yet been examined. The current study aims to investigate the effect and mechanism of the RNA methyltransferase METTL16 in CCA.

**Methods:**

The expression of METTL16 in CCA was examined by analyzing publicly available datasets or by IHC staining on tumor samples. siRNA or CRISPR/Cas9-mediated loss of function studies were performed in vitro and in vivo to investigate the oncogenic role of METTL16 in CCA. MeRIP-Seq was carried out to identify the downstream target of METTL16. ChIP-qPCR, immunoprecipitation, and immunoblots were used to explore the regulation mechanisms for METTL16 expression in CCA.

**Results:**

We observed that the expression of METTL16 was noticeably increased in human CCA tissues. Depletion of METTL16 significantly inhibited CCA cell proliferation and decreased tumor progression. PRDM15 was identified as a key target of METTL16 in CCA cells. Mechanistically, our data showed that METTL16 regulated PRDM15 protein expression via YTHDF1-dependent translation. Accordingly, we observed that restoration of PRDM15 expression could rescue the deficiency of CCA cell proliferation/colony formation induced by METTL16 depletion. Our subsequent analyses revealed that METTL16-PRDM15 signaling regulated the expression of FGFR4 in CCA cells. Specifically, we observed that PRDM15 protein was associated with the FGFR4 promoter to regulate its expression. Furthermore, we showed that the histone acetyltransferase p300 cooperated with the transcription factor YY1 to regulate METTL16 gene expression via histone H3 lysine 27 (H3K27) acetylation in CCA cells.

**Conclusions:**

This study describes a novel METTL16-PRDM15-FGFR4 signaling axis which is crucial for CCA growth and may have important therapeutic implications. We showed that depletion of METTL16 significantly inhibited CCA cell proliferation and decreased tumor progression.

**Supplementary Information:**

The online version contains supplementary material available at 10.1186/s13046-023-02844-5.

## Background

Cholangiocarcinoma (CCA) is a highly malignant cancer of the biliary system with a poor prognosis [1–5]. It is a lethal malignant tumor for which conventional anticancer therapies are often unsuccessful. Due to being diagnosed at an advanced stage, CCA patients have a 5-year overall survival rate of lower than 20% [6]. Thus, there is a crucial need to better understand the molecular mechanisms of cholangiocarcinogenesis to develop more effective target therapy.

The molecular mechanisms underlying CCA development and progression involve genetic and epigenetic changes leading to alterations of oncogenic and tumor suppressive pathways. Studies with integrative genomics approaches have led to the identification of dysregulated transcriptomic landscapes in CCA [7, 8]. The recent discovery of targetable genetic abnormalities in CCA patients promises a new era of precision medicine for this disease. For example, FGFRs 1–4 mutations, particularly FGFR2 fusion and FGFR4 overexpression, are often identified in CCA [9, 10]. In contrast to FGFR2 translocations, which have been linked to a better prognosis, FGFR4 overexpression independently predicts worse survival in CCA [11]. Accordingly, studies have shown that inhibition of FGFR4 suppresses CCA cell proliferation and invasion [10]. However, the mechanisms underlying the dysregulation of FGFR4 in CCA are incompletely defined.

Epigenetic modifications, such as DNA and histone alterations, are critical in the development and progression of human malignancies. Aside from DNA and histone modifications, recent evidence has pointed toward the involvement of RNA alterations in the regulation of gene expression and carcinogenesis. Notably, N6-Methyladenosine (m6A) modification is the most abundant modification in mRNA. This modification is catalyzed by m6A methyltransferases which catalyze the transfer of a methyl group (–CH3) to a hydrogen atom (–H) connected to the sixth nitrogen atom (N6). Several m6A binding proteins, including IGF2BP1/2/3 and the YTH family proteins, have been identified as m6A readers to regulate downstream effects. Among the m6A readers, YTHDF1 and YTHDF3 are known to regulate the translation of mRNAs [12, 13].

Although the m^6^A methyltransferase complex composed of METTL3 and METTL14 has been considered the main m^6^A writer [14–16], depletion of METTL3 and METTL14 results in a decrease of only approximately 60% in m^6^A modifications in some cell types, and a substantial portion of m^6^A marks in cells do not map well with the binding sites of METTL3/METTL14 [14, 17]. These observations suggest the existence of additional m^6^A methyltransferases. Notably, METTL16 is a recently identified m6A methyltransferase that has been shown to mediate m6A modification in a number of mRNA substrates [18–23]. The biological roles of METTL16 are just beginning to be understood, as they have not been fully investigated in comparison to METTL3/METTL14. While METTL16 was initially recognized as an essential factor for mouse embryonic development by regulating Mat2a mRNA expression [21], subsequent evidence has shown the involvement of METTL16 in the progression of several cancers, including gastric cancer, hepatocellular carcinoma, and breast cancer [18, 24–26]. A recent study shows that METTL16 expression is elevated in pancreatic ductal adenocarcinoma (PDAC) and correlated with better clinical outcomes for patients [27]. Wei et al. reports upregulated expression of METTL16 in cholangiocarcinoma [28], although the effect and mechanism of METTL16 in CCA remain largely unknown.

The current study was designed to investigate the effect and mechanism of METTL16 in CCA. We have analyzed the expression of METTL16 in several human CCA cohorts and observed that the expression of METTL16 is significantly upregulated in human CCA tissues. The impact of METTL16 on CCA cell growth was evaluated by complementary gene knockdown studies in vitro and in mice. Detailed mechanistic studies were performed to delineate the mechanisms of METTL16 actions in CCA cells. Our experimental findings disclose a novel METTL16-PRDM15-FGFR4 signaling axis which is crucial for CCA growth and may have important therapeutic implications.

## Materials and methods

### Cell culture

Human cholangiocarcinoma cell lines (CCLP1 and HuCCT1) were cultured according to the procedures we previously described [29]. Briefly, the cells were cultured in DMEM medium supplemented with 10% FBS and 1% antibiotics at 37 °C. The cells underwent authentication through short tandem repeat analysis and were consistently checked for mycoplasma contamination using the LookOut Mycoplasma PCR Detection Kit (Sigma-Aldrich) as part of routine certification procedures. For CRISPR/Cas9-mediated deletion of METTL16 in CCA cells, two guide RNAs were inserted into the lentiCRISPR V2 vector (#52,961, Addgene). The CRISPR plasmids were confirmed by Sanger sequencing. To generate lentivirus particles, 3 µg lentiCRISPR vectors, 0.75 µg pMD2.G (#12,259, Addgene) and 2.25 µg psPAX2 (#12,260, Addgene) were mixed well and co-transfected into HKE293T cells in a 60 mm dish with lipofectamine 3000. The lentivirus particles were collected 48 and 72 h post transfection and then added to CCLP1 and HuCCT1 with 5 µg/mL polybrene (sc-134220, Santa Cruz Biotechnology). After 48 h of infection, CCLP1 and HuCCT1 cells were passaged and selected using 1 µg/mL puromycin (A1113803, Gibco) for 1 week. Polyclonal METTL16 knockout cells were used to avoid the effects of single-clone selection.

### Cell proliferation and colony formation assays

For cell proliferation assay, 1 × 10^3^ CCA cells were seeded into 96-well plates. The WST-1 solution (CellPro-Ro, Roche) was applied to each 96-well plate at the given time points and incubated for 2 h. A microplate reader set at 450 nm was used to measure the absorbance. For colony formation assay, cells were seeded at a density of 500 cells/ well in 12-well plates and cultured for 7–14 days. After which colonies were stained with 0.5% crystal violet after treatment with a 4% paraformaldehyde solution. ImageJ software was used to count the colonies.

### RNA extraction and qRT-PCR

Total RNA was extracted from CCA cells using TRIZOL reagent (Invitrogen) according to the manufacturer’s instructions. The cDNA was synthesized using iScript™ Reverse Transcription Supermix (Bio-Rad). To confirm the expression of individual mRNAs, a quantitative real-time polymerase chain reaction (qRT-PCR) was performed using a CFX96 TOCH real-time system (Bio-Rad) with SYBR (Bio-Rad). The primer sequences used for qRT-PCR are shown in Supplementary Table S1.

### Western blot assay

Total protein extracts were isolated with Radioimmunoprecipitation assay (RIPA) buffer in the presence of protease and phosphatase inhibitor cocktail (Roche). Equal quantities of protein were separated using sodium dodecyl sulfate–polyacrylamide gel electrophoresis (SDS-PAGE), transferred to nitrocellulose membranes, immunoblotted with the appropriate antibodies, and revealed using Odyssey. The antibodies used in this study were as follows: METTL16 (Cell Signaling Technology, #87,538), PRDM15 (Proteintech, 25,590–1-AP), SETD5 (Abclonal, A7304, RRID:AB_2767845), KMT2D (Proteintech, 27,266–1-AP), NSD2 (Abclonal, A7938, RRID:AB_2772896), FGFR4 (Proteintech, 67,800–1-Ig), YTHDF1 (Proteintech, 17,479–1-AP, RRID:AB_2217473), YTHDF3 (Cell Signaling Technology, #24,206), FLAG (Cell Signaling Technology, #14,793), HA (Cell Signaling Technology, #3724), H3K27ac (Cell Signaling Technology, #8173), p300 (Cell Signaling Technology, #54,062), YY1 (Cell Signaling Technology, #46395S), β-actin (Sigma, A2228), and the anti-rabbit and anti-mouse secondary antibodies (LI-COR, Ride® 800CW).

### siRNA and GapmeR ASO transfections

Control siRNAs or siMETTL16, siPRDM15, siMETTL3, siYTHDF1, and siYTHDF3 siRNAs were obtained from Integrated DNA Technologies (IDT). The locked nucleic acid GapmeR antisense oligonucleotide (ASO) against METTL16 (5’-GCTTACTTGGTGGTGA-3’) was synthesized at QIAGEN. CCA cells were seeded into 6-well plates and cultured overnight. 50 nM siRNA or GapmeR ASO was transfected using Lipofectamine 3000 (Invitrogen, L3000015) following the manufacturer’s protocols.

### Immunochemistry (IHC) staining of CCA tissue

The human CCA tissue microarray was purchased from Pantomics (LVC1261). Following antigen retrieval, the tissue slides were incubated with primary antibodies against METTL16 (Abclonal, A15894), PRDM15 (Thermo Fisher Scientific, PA5-55,036, RRID: AB_2645923), and FGFR4 (Proteintech, 11,098–1-AP). After rinsing with TBS, the slide was incubated for 1 h at room temperature with a horseradish peroxidase-conjugated second antibody. After washing with TBS, the slide was incubated for 5 min at room temperature with 3,3′-diaminobenzidine (DAB) for chromogenic development. The IHC score was determined by multiplying the staining intensity (negative: 0; mild: 1; moderate: 2; severe: 3) with the staining area (negative: 0; ≤ 30%: 1; > 30% and ≤ 60%: 2; > 60%: 3).

### RNA immunoprecipitation (RIP)

The Magna RIP RNA-Binding Protein Immunoprecipitation Kit (Millipore) was used for RNA immunoprecipitation according to the manufacturer's procedure. Protein A/G magnet beads were coated with 10 µg of YTHDF1 antibody or normal IgG and treated with cell lysates overnight at 4 °C. The precipitated RNA was then separated using elution buffer, purified using Phenol/Chloroform/Isoamyl alcohol (25:24:1), and submitted to quantitative RT-PCR analysis. The primer sequences used for RIP-qPCR are shown in Supplementary Table S1.

### m6A sequencing

TRIzol reagent (Invitrogen) was used to extract total RNA from CCA cells. Subsequently, we performed mRNA-seq and m6A-seq simultaneously (LC Sciences, LLC, TX). For mRNA-seq, 1 μg of total RNA from CCA cells was subjected to poly(A) mRNA isolation with Dynabeads Oligo-dT (Thermo Fisher, cat.25–61,005). Then, a cDNA library was constructed following the TruSeq RNA Library Prep Kit v2 (Illumina) protocol. The library was subjected to 2 × 150 bp paired-end (PE150) sequencing on an Illumina NovaSeq 6000. For m6A-seq, the cleaved RNA fragments were incubated with the m6A antibody in immunoprecipitation buffer (750 mM NaCl, 50 mM Tris–HCl, and 0.5% Igepal CA-630), followed by PE150 sequencing on an Illumina NovaSeq™ 6000.

### Ribosomal immunoprecipitation

For ribosome immunoprecipitation, CCLP1 cells were transfected with control and siYTHDF1 for 48 h using Lipofectamine 3000. After which, an RPL22-FLAG construct (Origene) was transfected into the cells. Two days later, the transfected cells were lysed with RIPA buffer. Same amount of lysates from control and siYTHDF1 CCLP1 cells was incubated with FLAG or IgG antibodies overnight. Then RNA immunoprecipitation was performed as described in the RIP section. The immunoprecipitated RNA was analyzed by qRT-PCR.

### Methylated RNA immunoprecipitation (MeRIP) qPCR

To detect m6A modifications of individual mRNAs, MeRIP was conducted according to the manufacturer's instructions using the EpiQuick CUT&RUN m6A RNA Enrichment (MeRIP) kit (P-9018, Epigentek). Briefly, 10 μg of mRNA from each group were incubated with 2 μg m6A or control antibodies and 4 μl of Affinity Beads for 1.5 h at room temperature with rotation. Then the RNA was treated with 10 μl of Nuclear Digestion Enhancer and 2 μl of Cleavage Enzyme Mix at room temperature for 4 min. After immunoprecipitation, the proteins were digested by proteinase K and the RNA was eluted by Elution buffer. The immunoprecipitated RNA was examined by qPCR analysis. The primer sequences used are shown in Supplementary Table S1.

### Chromatin immunoprecipitation (ChIP) assay

The SimpleChIP® Plus Sonication Chromatin IP Kit (Cell Signaling Technology, #56,383) was used for the ChIP assay. In brief, cells were fixed in 1% formaldehyde and quenched in 125 mM glycine. Nuclei were extracted and sonicated to produce 200 bp fragments. 100 μl of the sonicated chromatin was immunoprecipitated overnight with 5 μg of the relevant antibodies for ChIP. Beads containing bound immunocomplexes were washed, rinsed, and DNA was extracted for qPCR. The primer sequences used are shown in Supplementary Table S1.

### In vivo mouse experiments

All animal studies were approved by the Institutional Animal Care and Use Committee of Tulane University. Six-week-old male NOD-SCID mice (NOD.Cg-*Prkdc*^*scid*^/J) were obtained from The Jackson Laboratory. For subcutaneous inoculation, 1 × 10^6^ control or METTL16-depleted CCA cells suspended in 100 µL PBS mixed with matrix gel (BD, 356,234) at 1:1 ratio was injected into the mice's flanks. Tumor sizes were measured at the relevant time intervals. At the end of the study, the mice were euthanized, and the tumors were dissected and weighed.

For intrahepatic inoculation, 1 × 10^6^ control or METTL16-depleted CCLP1 cells suspended in 10 µL PBS mixed with matrix gel (BD, 356,234) at a 1:1 ratio was implanted into the livers of NOD-SCID mice. The mice were sacrificed 8 weeks after injection, their livers were surgically dissected, and the liver/body weight ratios were evaluated.

### Statistical analysis

The data were analyzed using GraphPad Prism 9 and presented as mean ± SD as specified. Two-tailed, unpaired Student's t-tests, one-way ANOVA, or two-way ANOVA was used to assess statistical significance. The log-rank test was used to determine the statistical significance of survival (http://r2.amc.nl). The experiments were performed at 2 or 3 independent times. P < 0.05 was considered statistically significant.

## Results

### METTL16 is upregulated in CCA

**Fig. 1:**
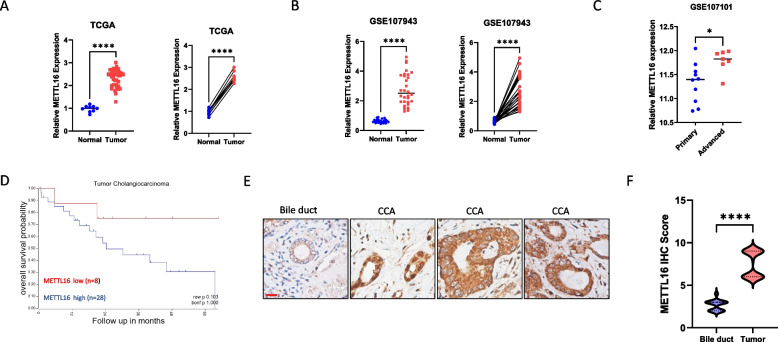
METTL16 is upregulated in CCA and correlates with poor prognosis. **A**, **B**. METTL16 expression in CCA tissues versus matched (left) or nonmatched (right) non-tumorous tissues from TCGA (**A**) and GSE107943 (**B**) datasets. **C**. METTL16 expression in 10 primary and 7 advanced CCA tumors in the GSE107101 dataset. **D**. Kaplan–Meier survival plot of CCA patients from the TCGA database stratified by low (red) and high (blue) METTL16 expression. **E**. Representative immunochemistry (IHC) staining images of METTL16 expression in the normal bile duct and CCA tissues. Scale bar: 10 µm. **F**. Quantitative result of IHC data as represented in E. *P < *0.05*, **P < *0.01*, ***P < *0.001*, ****P < *0.0001*. **A**-**C**, **E** Unpaired t-test

We have analyzed the expression of METTL16 in CCA and non-tumorous tissue samples from TCGA (https://www.tcga.org) and GEO (https://www.ncbi.nlm.nih.gov/geo) databases. Our analyses indicate that METTL16 is upregulated in CCA tissues when compared to matched or unmatched non-tumorous tissues in TCGA-Cholangiocarcinoma (TCGA-CHOL) (Fig. 1A) and GSE107943 (Fig. 1B) datasets. Analysis of GSE107101 dataset shows that advanced CCA tissues express higher level of METTL16 when compared to primary CCA tissues (Fig. 1C). Further survival analysis of patients with available clinical data from the TCGA-CHOL dataset shows that high expression of METTL16 in CCA tissues tends to be associated with worse patient survival (Fig. 1D). By performing immunohistochemistry (IHC) staining for METTL16 in human CCA tissues, we observe that the expression of METTL16 in CCA is significantly higher than in normal bile duct epithelium (Fig. 1E and F). These clinical analyses suggest a potential oncogenic role of METTL16 in CCA.

### Depletion of METTL16 inhibits CCA cell growth in vitro and in vivo

**Fig. 2:**
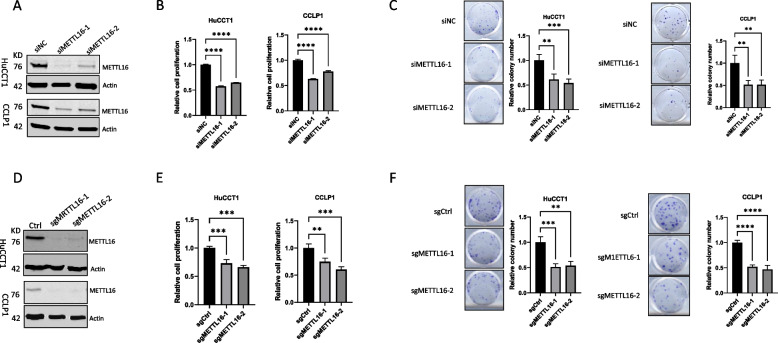
Depletion of METTL16 inhibits CCA cell proliferation and reduces clonogenicity, in vitro. Human CCA cells (HuCCT1 and CCLP1) were transfected with siRNAs targeting METTL-16 (siMETTL16-1 and siMETTL16-2) and control siRNA (siNC) or transfected with CRISPR/Cas-9 vector carrying METTL16-specific guide RNAs (sgMETTL16-1 or sgMETTL16-2). **A** Western blot for METTL16 in CCA cells transfected with control (siNC) or METTL16 targeting siRNAs. **B** WST-1 cell proliferation assay in CCA cells transfected with control (siNC) or METTL16 targeting siRNAs. **C** Colony formation assay in CCA cells transfected with control (siNC) or METTL16 targeting siRNAs. **D** Western blot for METTL16 in CCA cells with or without CRISPR/Cas9-mediated METTL16 deletion. **E** WST-1 cell proliferation in CCA cells with or without CRISPR/Cas9-mediated METTL16 deletion. **F** Colony formation assay in CCA cells with or without CRISPR/Cas9-mediated METTL16 deletion. **P < *0.01*, ***P < *0.001*, ****P < *0.0001*. **B**, **C**, **E**, **F** Mean ± SD, One-way ANOVA test

To determine the functional role of METTL16 in CCA cells, we used two sets of siRNAs to knock down METTL16 in two human CCA cell lines (CCLP1 and HuCCT1). Satisfactory knockdown efficacy was confirmed by Western blot analysis (Fig. 2A). We observed that knockdown of METTL16 led to a significant decrease in CCA cell proliferation and colony formation capability (Fig. 2B and C). In parallel, we had used the CRISPR/Cas9 gene editing approach to delete METTL16 expression in CCLP1 and HuCCT1 cells (Fig. 2D). Consistent with the siRNA knockdown results, METTL16 deletion by CRISPR/Cas9 also significantly reduced CCA cell proliferation and colony formation (Fig. 2E and F). These results demonstrate an important role of METTL16 for CCA cell growth, in vitro.

**Fig. 3:**
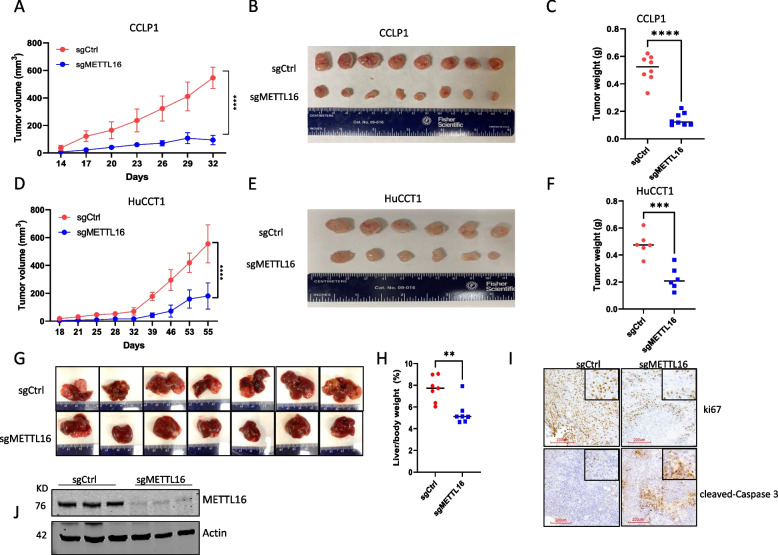
Depletion of METTL16 inhibits CCA growth in mice. (A-C) CCLP1 cells transfected with CRISPR/Cas-9 vector carrying METTL16-specific guide RNA (sgMETTL16-2) or control guide RNA (sgCtrl) were subcutaneously inoculated into SCID mice. Tumor volumes measured at indicated time points are shown in (**A**). Gross photographs of tumors recovered from mice sacrificed at the end time point are shown in (**B**). Tumor weights from control vs METTL16-depleted group are shown in (**C**). **D-F** HuCCT1 cells transfected with CRISPR/Cas-9 vector carrying METTL16-specific guide RNA (sgMETTL16-2) or control guide RNA (sgCtrl) were subcutaneously inoculated into SCID mice. Tumor volumes (**D**), gross photos (**E**), and tumor weights **F** are shown. (G-J) CCLP1 cells transfected with CRISPR/Cas-9 vector carrying METTL16-specific guide RNA (sgMETTL16-2) or control guide RNA (sgCtrl) were inoculated into the livers of SCID mice. The mice were sacrificed 6 weeks post-inoculation. Gross photographs of livers recovered from each group are shown in (**G**). The liver/body weight ratios of mice from each group are shown in (**H**). The expression of the cell proliferation marker Ki67 and the apoptosis marker cleaved caspase-3 was determined by IHC analysis (**I**). CRISPR/Cas9-mediated depletion of METTL16 in the tumor tissues recovered from mouse livers was confirmed by Western blotting (**J**). **P < *0.01,* ***P < *0.001,* ****P < *0.0001.* **A**, **D** Mean ± SD, One-way ANOVA test. **C**, **F**, **H** Unpaired t-test

We then inoculated CRISPR/Cas9-mediated METTL16 knockout and control CCA cells subcutaneously into NOD/SCID mice (NOD.Cg-*Prkdc*^*scid*^/J). As shown in Fig. 3A through F, depletion of METTL16 significantly inhibited CCA growth in this xenograft tumor model. In parallel, we had employed a separate CCA xenograft model in which human CCA cells were inoculated into the livers of NOD/SCID mice; we observed that deletion of METTL16 by CRISPR/Cas9 also significantly inhibited CCA growth when the tumor cells were inoculated into the livers (Fig. 3G-I). Together, these findings demonstrate an important role of METTL16 for CCA growth, in vivo.

### PRDM15 is a direct target of METTL16 in CCA cells

**Fig. 4:**
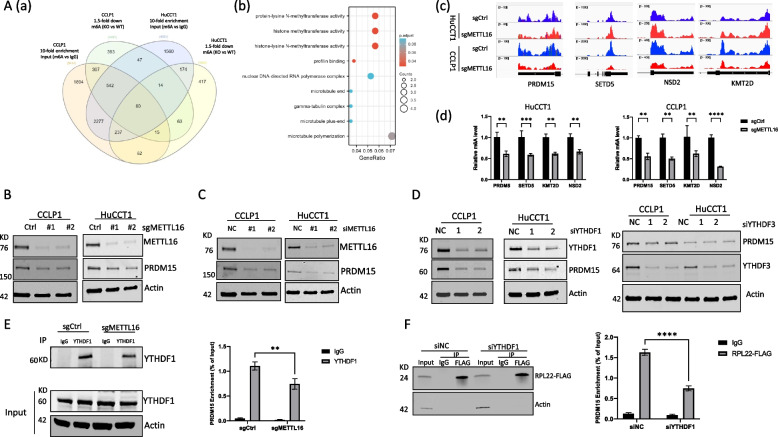
MeRIP-seq analysis identifies PRDM15 as a downstream target of METTL16. **A** (**a**) Venn diagram displays genes identified by MeRIP-seq from METTL16 knockout (KO, by guide RNA #2) and control cells (WT). **b** Gene ontology analysis of the 80 identified genes from A. **c** Integrative genomics viewer (IGV) plots show m6A peaks at PRDM15, SETD5, KMT2D, and NSD2 mRNAs in MeRIR-seq of HuCCT1 and CCLP1 cells. Ranges of reads are indicated. (**d**) MeRIP-qPCR analysis of m6A level in PRDM15, SETD5, KMT2D, and NSD2 mRNAs. **B**, **C** Depletion of METTL16 by CRISPR/Cas9 (**B**) or siRNA (**C**) decreases PRDM15 protein expression in CCLP1 and HuCCT1 cells as determined by Western blotting analysis. **D** Depletion of YTHDF1 reduces PRDM15 protein expression in CCA cells. CCLP1 and HuCCT1 cells were transfected with control or YTHDF1/YTHDF3 siRNAs for 48 h. The protein level of PRDM15 was measured by immunoblotting. **E** Depletion of METTL16 decreases the interaction between YTHDF1 protein and PRDM15 mRNA. YTHDF1 protein was immunoprecipitated from control or METTL16 knockout CCLP1 cells and analyzed by immunoblotting (left). The enrichment of PRDM15 mRNA in YTHDF1-RIP over IgG in control or METTL16 knockout CCLP1 cells was analyzed by RIP-qPCR (right). **F** Depletion of YTHDF1 decreases ribosome-bound PRDM15 mRNA. Immunoblotting analysis of immunoprecipitated RPL22-FLAG in CCLP1 cells transfected with control or YTHDF1 siRNA (left). Ribosomal immunoprecipitation assay was performed in siYTHDF1 and siNC CCLP1 cells transfected with RPL22-FLAG plasmid. **P < *0.01*, ***P < *0.001*, ****P < *0.0001*. **d**, **E**, **F** Mean ± SD, Two-way ANOVA test

To identify potential downstream targets responsible for the oncogenic role of METTL16 in CCA, we performed m6A methylated RNA immunoprecipitation sequencing (MeRIP-seq) in human CCA cells (CCLP1 and HuCCT1) with or without CRISPR/Cas9-mediated METTL16 depletion. By analyzing the m6A RNA-seq data, we identified 80 genes with m6A RNA modification (tenfold enrichment compared to IgG) that were decreased at least 1.5-fold in these two cell lines upon METTL16 knockout (Fig. 4A(a), Supplementary Table S2). Gene Ontology (GO) enrichment analysis of the identified genes revealed an enrichment in biological processes involving methyltransferase activity (Fig. 4A(b)). Among the identified genes are PRDM15, SETD5, KMT2D and NSD2, which are all upregulated in CCA when compared to normal tissues (Supplementary Fig. 1). The reads of m6A peaks mapping to these 4 genes were decreased in METTL16-deleted cells when compared to control cells (Fig. 4A(c)). MeRIP-qPCR confirmed that their m6A levels were significantly reduced after METTL16 deletion in CCLP1 and HuCCT1 cells (Fig. 4A(d)).

We then measured the mRNA levels of the identified four genes (PRDM15, SETD5, KMT2D and NSD2) by qRT-PCR in CCLP1 and HuCCT1 cells with or without METTL16 depletion. Our data showed that deletion of METTL16 did not affect their mRNA levels (Supplementary Fig. 2). Western blotting analysis showed that METTL16 depletion reduced the protein expression of PRDM15, while it did not affect the protein levels of SETD5, KMT2D and NSD2 (Fig. 4B, C, and Supplementary Fig. 3). Thus, PRDM15 represents a direct target of METTL16 in CCA cells.

As METTL3/METTL14 complex has been considered as a main m6A writer [14, 30], we sought to examine whether PRDM15 mRNA might also be methylated by this complex. To this end, we had depleted METTL3 expression in CCA cells and measured m6A modification of PRDM15 mRNA (note that METTL14 is undetectable in CCA cells). Our results showed that knockdown of METTL3 did not influence m6A modification of PRDM15 mRNA (Supplementary Fig. 4A). Additionally, knockdown of METTL3 exhibited minimal effect on PRDM15 protein expression (Supplementary Fig. 4B). These results support the notion that PRDM15 is a specific target of METTL16, but not METTL3, in CCA cells.

### METTL16 regulates PRDM15 expression through YTHDF1-dependent translation mechanism in CCA cells

Our findings that depletion of METTL16 reduces the level of PRDM15 protein but not mRNA suggest that METTL16 may regulate PRDM15 protein expression through translational modulation. In this context, YTHDF1 and YTHDF3 are known to be the key molecules among m6A readers for regulation of translation [12, 13]. We then performed further studies to determine whether these two m6A reader proteins might be implicated in the translation of PRDM15 mRNA. Our data showed that knockdown of YTHDF1, but not YTHDF3, decreased PRDM15 protein expression in CCA cells (Fig. 4D). These findings suggest the role of YTHDF1 for regulation of PRDM15 protein expression in CCA cells.

Based on the above results, we subsequently performed RNA immunoprecipitation (RIP) experiments to determine whether YTHDF1 protein might interact with PRDM15 mRNA. Our data showed that YTHDF1 protein was able to bind PRDM15 mRNA in CCA cells. Notably, depletion of METTL16 significantly decreased the interaction between YTHDF1 protein and PRDM15 mRNA (Fig. 4E). These findings suggest that the m6A reader protein YTHDF1 binds to PRDM15 mRNA in a manner dependent on METTL16-mediated m6A modification.

To further determine the role of YTHDF1 in PRDM15 translation, we performed ribosome immunoprecipitation assay in CCA cells that were transfected with FLAG-tagged ribosome protein RPL22. In this assay, mRNA transcripts isolated from ribosomes were analyzed by qRT-PCR to quantify PRDM15 mRNA. As shown in Fig. 4F, while ribosomes accumulated PRDM15 mRNA in control cells, the ribosome occupancy of PRDM15 mRNA was significantly decreased in cells with YTHDF1 depletion. Together, these results suggest that METTL16 regulates PRDM15 via RNA m6A modification and YTHDF1-dependent translation mechanism in CCA cells.

### The effect of PRDM15 on CCA cell proliferation and clonogenicity

**Fig. 5:**
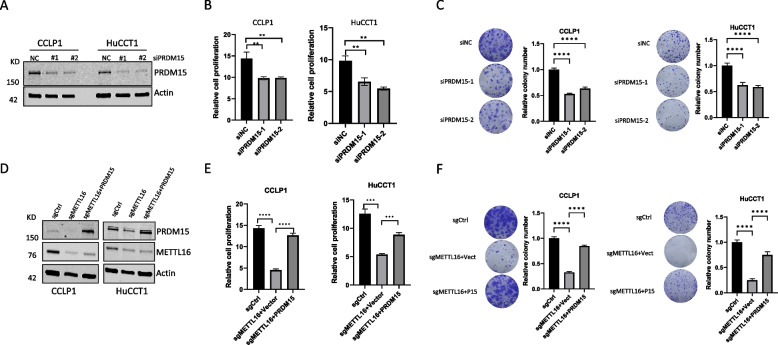
PRDM15 is essential for METTL16-mediated CCA cell growth. **A**-**C** Depletion of PRDM15 inhibits CCA cell proliferation and colony formation capability. The efficacy of PRDM15 depletion by siRNA was validated by immunoblotting analysis (**A**). WST-1 cell proliferation (**B**) and colony formation (**C**) assay were performed in CCLP1 and HuCCT1 cells transfected with siPRDM15 and control siRNA. **D-F** Forced overexpression of PRDM15 rescues the deficiency of CCA cell proliferation and colony formation induced by METTL16 depletion. PRDM15 expression construct was transfected in CCLP1 and HuCCT1 cells for 48 h and PRDM15 protein expression was determined by immunoblotting (D). WST-1 cell proliferation (**E**) and colony formation (**F**) assay were performed in CCLP1 and HuCCT1 cells transfected with PRDM15 overexpression or control construct. **P < *0.01*, ***P < *0.001*, ****P < *0.0001*. **B**, **C**, **E**, **F** Mean ± SD, One-way ANOVA test

While PRDM15 has been shown to play a role in stem cell biology and during early development [31], its role in cancer biology remains undefined. Up to now, no study has been conducted to examine the role of PRDM15 in CCA. Therefore, we performed immunohistochemical staining for PRDM15 in human CCA tissues. Our data showed a high expression of PRDM15 in CCA tissues compared to the negative staining in bile duct epithelium (Supplementary Fig. 5A and B). Consistent with regulation of PRDM15 by METTL16, we observed a positive correlation between PRDM15 and METTL16 in CCA tissues (Supplementary Fig. 5C). To further document the functional role of PRDM15 in CCA, we analyzed the cell proliferation and colony formation capability in CCA cells with PRDM15 depletion. Our data showed that depletion of PRDM15 significantly inhibited CCA cell proliferation and colony formation capability (Fig. 5A-C). Based on these results, we then performed rescue experiments with forced overexpression of PRDM15 in METTL16-depleted CCA cells. We observed that restoration of PRDM15 was able to partially rescue the deficiency of CCA cell proliferation/colony formation induced by METTL16 depletion (Fig. 5D**-**F). These results demonstrate that PRDM15 is a functionally important target of METTL16 in CCA cells.

### METTL16-induced PRDM15 regulates the expression of FGFR4 in CCA cells

**Fig. 6:**
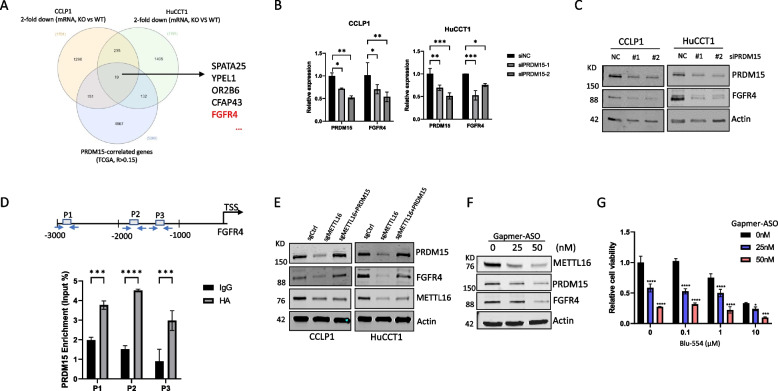
PRDM15 regulates FGFR4 expression by binding its promoter. **A** Venn diagram displays METTL16-regulated genes (from RNA-seq data in CCLP1 and HuCCT1 cells with or without METTL16 knockout) and genes that positively correlated with PRDM15 in CCA from the TCGA database. **B** Depletion of PRDM15 decreased the mRNA level of FGFR4. RT-qPCR analysis of FGFR4 expression in CCLP1 and HuCCT1 cells transfected with siPRDM15 or siNC. **C** Depletion of PRDM15 decreased the protein level of FGFR4. CCLP1 and HuCCT1 cells were transfected with siPRDM15 or siNC for 48 h. FGFR4 protein expression was measured by immunoblotting. **D** Putative binding sites of PRDM15 in the promoter region of FGFR4 (top). ChIP-qPCR was performed in CCLP1 cells transfected with PRDM15-HA construct (bottom). **E** Forced overexpression of PRDM15 reversed FGFR4 expression in METTL16-depleted cells. CCLP1 and HuCCT1 cells were transfected with the PRDM15 expression construct for 48 h. PRDM15 and FGFR4 protein expression was determined by immunoblotting. **F** Inhibition of METTL16 by next generation GampeR ASO reduced PRDM15 and FGFR4 protein expression. CCLP1 cells were transfected with METTL16-specific GampeR ASO for 72 h. The levels of PRDM15 and FGFR4 proteins were determined by immunoblotting. **G** Depletion of METTL16 by next generation GampeR ASO enhances the tumor inhibitory effect of the FGFR4 inhibitor BLU-554. CCLP1 cells were transfected with METTL16 GampeR ASO and treated with the FGFR4 inhibitor BLU-554 for 72 h. Cell viability was determined by WST1 assay. *P < 0.05, **P < 0.01, ***P < 0.001, ****P < 0.0001. Mean ± SD, Two-way ANOVA test in **B**, **D**, and **G**

As PRDM15 is a transcriptional regulator, we sought to further identify the potential downstream target of METTL16-induced PRDM15 in CCA cells. For this purpose, we compared the genes that are regulated by METTL16 in CCA cells (from our RNA-Seq data) with the genes that are positively correlated with PRDM15 (from the TCGA-CHOL database). This approach led to the identification of 19 METTL16-regulated genes which were positively correlated with PRDM15 in CCA (Fig. 6A, Supplementary Table S3). Among the 19 identified genes, we elected to focus on FGFR4 which has been well-documented to play an important role in CCA [10, 32, 33]. To determine the effect of PRDM15 on FGFR4 expression, we analyzed the mRNA and protein expression of FGFR4 in CCA cells with PRDM15 depletion. Our data showed that depletion of PRDM15 decreased both the mRNA and protein levels of FGFR4 (Fig. 6B and C). Next, we evaluated whether FGFR4 might be a direct target of PRDM15. To this end, we performed transcription factor binding profile analysis (using JASPAR database) to search for possible PRDM15 binding site in the FGFR4 gene promoter. This effort led to the identification of three putative PRDM15 binding sites in the FGFR4 promoter (illustrated in Fig. 6D). Based on this information, we carried out ChIP-qPCR assays in CCA cells transfected with HA-tagged PRDM15 to determine its association with the FGFR4 gene promoter. Indeed, our experimental results confirmed the binding of PRDM15 to the promoter of the FGFR4 gene in CCA cells (Fig. 6D). To further validate the regulation of FGFR4 by METTL16-PRDM15, we examined FGFR4 expression in METTL16 depleted cells with or without PRDM15 restoration. Our data showed that depletion of METTL16 led to a decrease in FGFR4 expression and that this effect was reversed by forced overexpression of PRDM15 (Fig. 6E). Furthermore, we observed a positive correlation between FGFR4 and METTL16 in CCA tissues (Supplementary Fig. 6A-C). Consistent with METTL16 mediated regulation of PRDM15-FGFR4, our data revealed that depletion of METTL16 by its GapmeR antisense oligonucleotide (ASO) decreased the protein levels of PRDM15 and FGFR4 in CCA cells (Fig. 6F). The above findings disclose a novel METTL16-PRDM15-FGFR4 signaling axis in CCA cells. We next performed recuse experiments with forced overexpression of FGFR4 in METTL16 depleted CCA cells. Our data showed that restoration of FGFR4 was able to partially rescue the deficiency of CCA cell proliferation and colony formation induced by METTL16 depletion (Supplementary Fig. 7A-C). Given that FGFR4 inhibitor treatment can prevent the growth of several types of cancer cells, we further evaluated the effect of FGFR4 inhibition in conjunction with METTL16 inhibition on CCA cell growth. While the FGFR4 inhibitor BLU-554 or the METTL16 GapmeR ASO alone was able to inhibit CCA cell growth, we found that combinational use of both the FGFR4 inhibitor BLU-554 and the METTL16 GapmeR ASO exhibited more tumor inhibitory effect (Fig. 6G). The latter results suggest an intriguing possibility of inhibiting METTL16 in conjunction with FGFR4 inhibitor for CCA treatment.

We further examined two FGFR4 downstream targets, ERK1/2 and AKT, in CCA cells. Our data showed that treatment of CCA cells with the FGFR4 inhibitor (BLU-554) decreased the phosphorylation of both ERK1/2 and AKT (S473) (Supplementary Fig. 7D). Similarly, depletion of METTL16 by CRISP-Cas9 also decreased the phosphorylation of ERK1/2 and AKT (S473) in CCA cells (Supplementary Fig. 7E). These findings suggest that ERK1/2 and AKT pathway represent potentially important downstream molecules of the METTL16-PRDM15-FGFR4 axis in CCA cells.

### Regulation of METTL16 expression by P300-mediated Histone H3 Lysine 27 acetylation (H3K27ac)

**Fig. 7:**
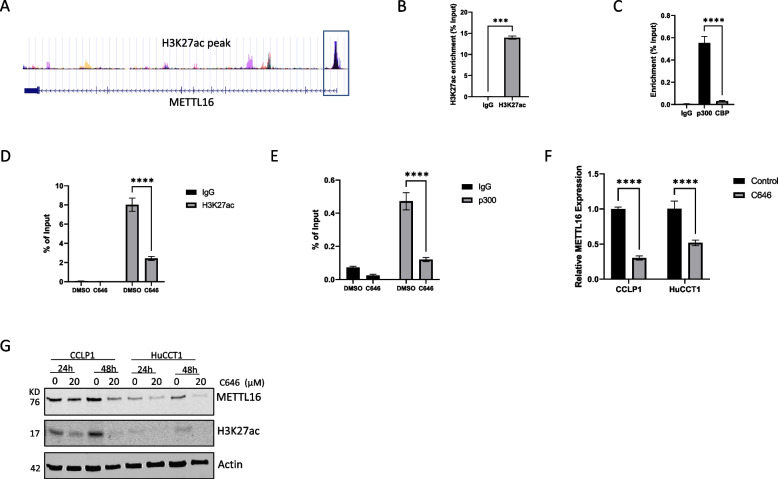
Regulation of METTL16 expression by p300-mediated H3K27ac in CCA. **A** Analysis of the UCSC Genome Browser (http://genome.ucsc.edu/) indicates that the promoter region of METTL16 is highly enriched with H3K27ac. **B** ChIP-qPCR assay using anti-H3K27ac revealed a gain of H3K27ac at the METTL16 promoter in CCLP1 cells. **C** ChIP-qPCR assay using anti-p300 and anti-CBP showed enrichment of p300 at the METTL16 promoter in CCLP1 cells. **D**, **E** Enrichment of H3K27ac (**D**) and p300 (**E**) at the promoter region of METTL16 was evaluated by ChIP-qPCR assay in CCLP1 cells treated with C646 (20 µM) for 48 h. **F** METTL16 mRNA expression level was determined by RT-qPCR in CCLP1 and HuCCT1 cells treated with C646 (20 µM) for 48 h. **G** METTL16 protein expression level was analyzed by immunoblotting in CCLP1 and HuCCT1 cells treated with C646 at indicated time points. ***P < *0.001*, ****P < *0.0001*. (B) Mean ± SD, Unpaired t-test. **C**-**F** Mean ± SD, Two-way ANOVA test

Given the noticeable increase of METTL16 expression and its oncogenic action in CCA, we carried out further studies to determine the mechanisms underlying the upregulation of METTL16 in CCA cells. We had analyzed the METTL16 promoter using the UCSC Genome Browser (https://genome.ucsc.edu/). This analysis revealed that the promoter region of METTL16 gene was highly enriched with histone H3 lysine 27 acetylation (H3K27ac) (Fig. 7A). This observation suggests that the expression of METTL16 may be controlled at the transcriptional level by histone acetylation. By performing ChIP-qPCR assays using anti-H3K27ac, we had identified a gain of H3K27ac at the METTL16 promoter in CCA cells (Fig. 7B). Since p300 (also known as EP300) and CBP are necessary for histone acetylation [34, 35], we performed separate ChIP-qPCR assays using anti-p300 and anti-CBP; results of these studies confirmed the enrichment of p300, but not CBP, at the METTL16 promoter (Fig. 7C). Based on these results, we performed further experiments by treating CCA cells with the p300 inhibitor, C646. Our data showed that treatment of CCA cells with the p300 inhibitor C646 significantly reduced the H3K27ac and p300 enrichment at the METTL16 promoter (Fig. 7D and E). Accordingly, C646 treatment also decreased METTL16 mRNA and protein levels in CCA cells (Fig. 7F and G). These findings demonstrate regulation of METTL16 expression by P300-mediated histone H3 lysine 27 acetylation in CCA cells.

### YY1 cooperates with p300 to regulate METTL16 expression in CCA cells

**Fig. 8:**
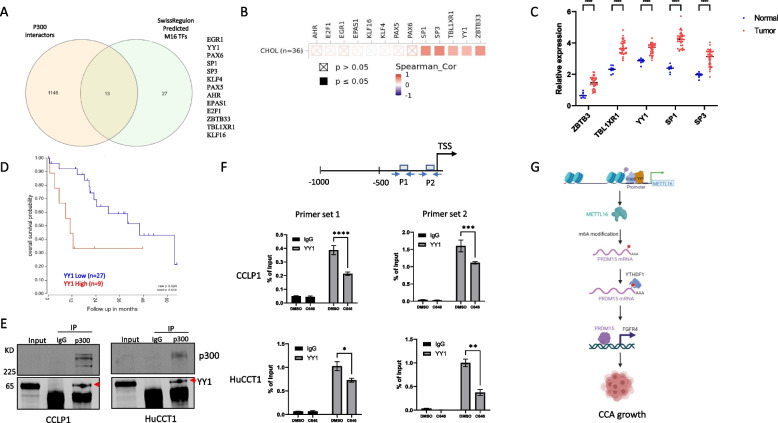
p300 cooperates with YY1 to regulate METTL16 expression. **A** Venn diagram displays overlapping genes of p300 interactors (BioGrid) and predicted transcription factors (TFs) for METTL16 (SwissRegulon). **B** Spearman correlation analysis of METTL16 and the identified TFs in CCA tissues from the TCGA database. **C** ZBTB3, TBLXR1, YY1, SP1, and SP3 expression in CCA and normal tissues from the TCGA database. **D** Kaplan–Meier survival plot of CCA patients from TCGA database stratified by low (blue) and high (red) YY1 expression. **E** The interaction between p300 and YY1 in CCLP1 and HuCCT1 cells was validated by immunoprecipitation assay. **F** The binding sites of YY1 on the promoter region of METTL16 were predicted by JASPAR (top). ChIP-qPCR was performed in CCLP1 and HuCCT1 cells treated with C646 (20 µM) for 48 h (bottom). **G** Proposed model for the roles of METTL16 in CCA. (Created using tools from BioRender) *P < *0.05*, **P < *0.01*, ***P < *0.001*, ****P < *0.0001*. **C**, **F** Mean ± SD, Two-way ANOVA test

As p300 is usually recruited by transcription factors (TFs) to target gene promoters [36, 37], we carried out further studies to identify the transcription factor(s) which may cooperate with p300 to regulate METTL16 expression in CCA cells. By analyzing p300 interactors predicted by BioGrid and putative transcription factors of METTL16 predicted by SwissRegulon, we detected 13 transcription factors common in these two datasets (Fig. 8A). Through correlation analysis between METTL16 and the identified transcription factors in CCA tissues from the TCGA database, we found that 5 of the TFs were positively correlated with METTL16 in CCA tissues, including SP1, SP3, TBL1XR1, YY1, and ZBTB3 (Fig. 8B). While the above five TFs were elevated in CCA compared to non-tumorous tissues (Fig. 8C), only the expression of YY1 correlated with patient survival in CCA (Fig. 8D). Based on these analyses, we performed further experiments to document the interaction between YY1 and P300 in CCA cells. As shown in Fig. 8E, immunoprecipitation assay confirmed association of p300 with YY1 in CCLP1 and HuCCT1 cells. Through analysis of the JASPAR database, we identified two YY1 binding sites in the promoter region of METTL16 (Fig. 8F, top panel). By performing ChIP-qPCR assays, we showed that YY1 was able to bind to these two sites at the METTL16 promoter. Furthermore, we observed that treatment of CCA cells with the p300 inhibitor C646 significantly reduced YY1 binding to the METTL16 promoter (Fig. 8F, bottom panel). Collectively, our findings indicate that YY1 cooperates with p300 to regulate METTL16 gene expression in CCA cells.

## Discussion

The current study provides novel evidence for an important role of METTL16 in CCA. Our data reveal that METTL16 is highly expressed in human CCA tissues and importantly implicated in the regulation of CCA cell growth. We show that PRDM15 is a direct downstream target of METTL16 in CCA cells. Our data indicate that METTL16 regulates PRDM15 protein expression via YTHDF1-dependent translation. We further show that METTL16-induced PRDM15 protein binds to the promoter of FGFR4 gene and enhances its expression in CCA cells. Our experimental results disclose a novel METTL16-PRDM15-FGFR4 signaling axis in CCA which may represent an effective therapeutic target. Moreover, our data reveal that the histone acetyl transferase p300 cooperates with the transcription factor YY1 to regulate METTL16 gene expression via histone H3 lysine 27 (H3K27) acetylation in CCA cells (illustrated in Fig. 8G).

Dysregulation of m6A modification and its machinery have been implicated in multiple types of cancer, including CCA. As core components of the m6A methyltransferase complex, METTL3/METTL14 have been linked to CCA progression [38, 39]. On the other hand, METTL16 is a newly discovered m6A methyltransferase that is not part of the METTL3/14 complex. According to high throughput cancer cell line CRISPR screenings, METTL16 appears to play a more important role in cancer cell survival and proliferation than METTL3 [40, 41]. However, prior to the current study, the potential role of METTL16 in CCA was not known. Consistent with the study by Wei et al. [28], we validated the upregulation of METT16 expression in CCA tissues compare to non-tumor tissues. Our experimental results described in this study establish an important oncogenic role of METTL16 in CCA and identify PRDM15 as a key downstream target of METTL16 via an m6A-dependent mechanism. Of note, the mechanisms for METTL16-regulated CCA cell growth appears to be different from those reported for some other cancer types. For example, METTL16 has been shown to drive leukemogenesis by promoting the expression of BCAT1 and BCAT2 in an m6A-dependent manner [22]. In pancreatic ductal cancer, METTL16 has been shown to inhibit MRE11-mediated DNA end resection and contributes synthetic lethality to PARP inhibition independent of m6A modification [27]. Therefore, the mechanisms mediating METTL16 actions in different cancer types are likely to be context dependent.

METTL3/METTL14, and METTL16 exhibit distinct crystal structures, which results in them having varied preferences for substrate when they recognize mRNA sequences directly [15, 16, 21]. Prior investigations indicate that METTL3 and METTL14 primarily identify single-stranded RNA that contains a RRACH sequence (R = G, A, and U; H = U, A, and C), whereas METTL16 tends to target structured RNA that carries a conserved UACAGAGAA motif [14, 20]. Our sequence analysis reveals the presence of the METTL16 binding motif in the PRDM15 mRNA. Together, our findings establish PRDM15 as a bona fide target of METTL16 in CCA.

PRDM15 is a member of the PRDF1 and RIZ1 homology domain-containing (PRDM) proteins which are sequence-specific transcriptional regulators involved in cell stemness and development, often dysregulated in cancer [42–45]. While studies have shown that PRDM15 is overexpressed in B-cell lymphomas and colon adenocarcinoma and contributes to tumorigenesis and radioresistance [43, 46], it remains unknow whether PRDM15 is implicated in CCA. Our results presented in this study show that PRDM15 is upregulated in CCA in comparison to non-tumorous tissues. The functional role of PRDM15 in CCA is attested by the observations that depletion of PRDM15 significantly inhibited CCA cell proliferation and colony formation capability. By performing rescue experiments with forced overexpression of PRDM15 in METTL16-depleted CCA cells, we observed that restoration of PRDM15 was able to partially rescue the deficiency of CCA cell proliferation/colony formation induced by METTL16 depletion. These findings support that PRDM15 is a functionally important target of METTL16 in CCA cells.

Our experimental results show that METTL16 upregulates the expression of PRDM15 protein through a mechanism involving METTL16-medicated m6A modification of PRDM15 mRNA that is recognized by the m6A reader protein YTHDF1 leading to increased PRDM15 translation. It is worth mentioning that the role of YTHDF1 in CCA is also corroborated by the documented effect of YTHDF1 on the translation of other oncogenic mRNAs (such as EGFR) [47].

Our further studies have led to the identification of FGFR4 as a downstream target regulated by METTL16-induced PFDM15. PRDM15 is a sequence-specific transcriptional regulator which directly binds to the AAAACCTGG motif located in the promoters of target genes. Our analysis reveals three putative PRDM15 binding sites in the FGFR4 promoter. By performing ChIP-qPCR assays in CCA cells transfected with HA-tagged PRDM15, we have demonstrated that the association of PRDM15 with its DNA biding sites in the FGFR4 promoter. Our separate studies indicate that depletion of PRDM15 in CCA cells reduces the mRNA and protein levels of FGFR4. Together, our experimental results demonstrate that FGFR4 is a direct target of PRDM15 in CCA.

FGFR4 overexpression is an important oncogenic alteration in CCA [9, 10, 48]. Notably, FGFR4 overexpression can independently predict worse survival in CCA patients [11, 49]. Functional studies have shown that inhibition of FGFR4 suppresses CCA cell proliferation and invasion [10]. However, the mechanisms underlying FGFR4 dysregulation in CCA remain to be further defined. Rizvi and colleagues have shown that the expression of FGFR4 in CCA cells is regulated by the oncogene YAP [50]. Our experimental results presented in this study reveal a new mechanism for FGFR4 expression regulated by METTL16-PRDM15 signaling in CCA.

FGFR4-selective inhibitors have been tested in clinical trials of patients with hepatocellular carcinoma (HCC), but the patient response to FGFR4-selective inhibitors in HCC appeared to be unsatisfactory [51]. To date, FGFR4 inhibitors have not yet been evaluated in CCA patients. In the current study, we observed that treatment with the FGFR4 inhibitor BLU-554 suppressed CCA cell survival in a dose-dependent manner. Meanwhile, our data showed that targeting METTL16 with specific GapmeR ASO was also able to inhibit CCA cell growth. Our data showed that depletion of METTL16 by GampeR ASO enhances the tumor inhibitory effect of the FGFR4 inhibitor BLU-554. This finding may suggest a potential strategy to enhance the efficacy of FGFR4-inhibitor in CCA treatment.

Thus far, the mechanisms underlying upregulation of METTL16 in cancer remain unexplored. Our findings in the current study disclose that the METTL16 promoter region is enriched with acetylated histone H3 lysine 27 (H3K27ac). Our results indicate that the histone acetyltransferase p300 plays an important role in histone H3 lysine 27 acetylation and transcriptional regulation of METTL16 gene expression in CCA cells. We discovered that p300 is recruited to METTL16 promoter by the transcription factor YY1. The latter assertion is based on the observations that YY1 directly interacts with p300 in CCA cells and can bind to the two YY1 binding sites located in the promoter region of the METTL16 gene. Our findings provide novel evidence that the histone acetyltransferase p300 cooperates with the transcription factor YY1 to regulate METTL16 gene expression in CCA cells. Together, our experimental results uncover an important epigenetic mechanism by which the p300/YY1 complex controls METTL16 expression via H3K27 acetylation.

In conclusion, the current study provides the first evidence that METTL16 is upregulated in CCA through an epigenetic mechanism which involves p300/YY1 complex and H3K27 acetylation. The oncogenic role of METTL16 is demonstrated by results from complementary in vitro and in vivo studies. Our experimental findings disclose a novel METTL16-PRDM15-FGFR4 signaling axis which is importantly implicated in CCA and may serve as a new therapeutic target.

### Supplementary Information

**Additional file 1: Supplementary Figure 1 **(related to Fig. 4). PRDM15, NSD2, KMT2D, and SETD5 expression in cholangiocarcinoma and normal tissues from the TCGA database. **Supplementary Figure 2** (related to Fig. 4). RT-qPCR analysis of PRDM15, NSD2, KMT2D and SETD5 expression in control and METTL16 knockdown cells. **Supplementary Figure 3** (related to Fig. 4). SETD5, KMT2D, and NSD2 protein expression were determined by an immunoblotting assay in control and METTL16 knockdown cells. **Supplementary Figure 4** (related to Fig. 4). A. MeRIP-qPCR assay was performed in METTL3 silencing (siMETTL3 #1) and control CCLP1 and HuCCT1 cells to detect the level of m6A-modified PRDM15 mRNA. B. PRDM15 protein expression was determined by an immunoblotting assay in control and METTL3 knockdown cells. **Supplementary Figure 5** (related to Fig. 5) . The expression of PRDM15 and METTL16 is positively correlated in human CCA tissues. (A). Representative immunochemistry (IHC) staining for PRDM15 in normal bile duct and CCA tissues. Scale bar: 100 μm. (B). Quantitative result of the IHC data as represented in A. (C). Positive correlation between PRDM15 and METTL16 expression in CCA patient samples (Pearson correlation coefficient analysis based on the IHC scores). **Supplementary Figure 6** (related to Fig. 6). The expression of FGFR4 is positively correlated with METTL16 in CCA tissues. (A). Representative immunochemistry (IHC) staining for FGFR4 expression in human CCA tissues and non-tumorous bile duct. Scale bar: 100 μm. (B). Quantitative result of the IHC data as represented in A. (C). Positive correlation between FGFR4 and METTL16 expression in CCA) patient samples (Pearson correlation coefficient analysis based on the IHC scores). **Supplementary Figure 7** (related to Fig. 6). Forced overexpression of FGFR4 rescues the deficiency of CCA cell proliferation and colony formation induced by METTL16 depletion. V5-tagged FGFR4 expression construct was transfected in CCLP1 and HuCCT1 cells for 48 hours and FGFR4 protein expression was determined by immunoblotting (A). WST-1 cell proliferation (B) and colony formation (C) assay were performed in CCLP1 and HuCCT1 cells transfected with FGFR4 overexpression or control construct. (D, E) Phosphorylation of FGFR4 signaling pathway components ERK1/2 and AKT were determined in BLU-554 treated (D) or METTL16 depletion (E) CCA cells by western blotting assay. **P<0.01, ***P<0.001, ****P<0.0001. (B, C, E, F) Mean ± SD, One-way ANOVA test. 

**Additional file 2: Table S1. **Primers used in this study.

**Additional file 3: Table S2. **Overlapping genes identified by m6A-seq from METTL16 knockout and control CCLP1 and HuCCT1 cells.

**Additional file 4: Table S3. **Overlapping genes identified from RNA-seq and TCGA database. 

## Data Availability

The data produced in this study can be found in the article and its supplementary data files and can also be obtained by contacting the corresponding author. The publicly available data used in this study, which were generated by other researchers, were sourced from TCGA-CHOL and Gene Expression Omnibus (GEO) databases under the accession numbers GSE107943 and GSE107101.
