# Supplementary material for: The RNA methyltransferase METTL16 enhances cholangiocarcinoma growth through PRDM15-mediated FGFR4 expression
Source: J Exp Clin Cancer Res. 2023 Oct 11;42:263. doi: 10.1186/s13046-023-02844-5 (PMC10566113; doi:10.1186/s13046-023-02844-5)
Supplement: Supplementary file 1 — Additional file 1: Supplementary Figure 1 (related to Fig. 4). PRDM15, NSD2, KMT2D, and SETD5 expression in cholangiocarcinoma and normal tissues from the TCGA database. Supplementary Figure 2 (related to Fig. 4). RT-qPCR analysis of PRDM15, NSD2, KMT2D and SETD5 expression in control and METTL16 knockdown cells. Supplementary Figure 3 (related to Fig. 4). SETD5, KMT2D, and NSD2 protein expression were determined by an immunoblotting assay in control and METTL16 knockdown cells. Supplementary Figure 4 (related to Fig. 4). A. MeRIP-qPCR assay was performed in METTL3 silencing (siMETTL3 #1) and control CCLP1 and HuCCT1 cells to detect the level of m6A-modified PRDM15 mRNA. B. PRDM15 protein expression was determined by an immunoblotting assay in control and METTL3 knockdown cells. Supplementary Figure 5 (related to Fig. 5) . The expression of PRDM15 and METTL16 is positively correlated in human CCA tissues. (A). Representative immunochemistry (IHC) staining for PRDM15 in normal bile duct and CCA tissues. Scale bar: 100 μm. (B). Quantitative result of the IHC data as represented in A. (C). Positive correlation between PRDM15 and METTL16 expression in CCA patient samples (Pearson correlation coefficient analysis based on the IHC scores). Supplementary Figure 6 (related to Fig. 6). The expression of FGFR4 is positively correlated with METTL16 in CCA tissues. (A). Representative immunochemistry (IHC) staining for FGFR4 expression in human CCA tissues and non-tumorous bile duct. Scale bar: 100 μm. (B). Quantitative result of the IHC data as represented in A. (C). Positive correlation between FGFR4 and METTL16 expression in CCA) patient samples (Pearson correlation coefficient analysis based on the IHC scores). Supplementary Figure 7 (related to Fig. 6). Forced overexpression of FGFR4 rescues the deficiency of CCA cell proliferation and colony formation induced by METTL16 depletion. V5-tagged FGFR4 expression construct was transfected in CCLP1 and HuCCT1 cells for 48 hours [file 13046_2023_2844_MOESM1_ESM.pdf]

SUPPLEMENTARY FIGURES

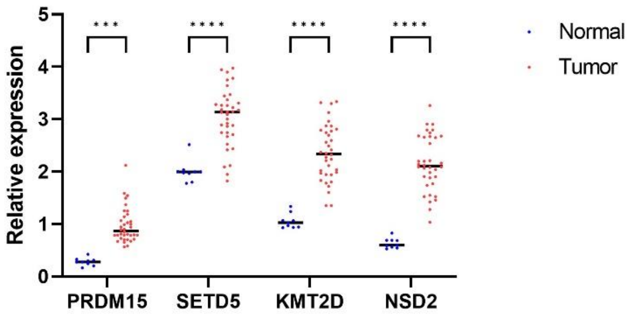

Supplementary Figure 1 (related to Figure. 4) PRDM15, NSD2, KMT2D, and SETD5 expression in cholangiocarcinoma and normal tissues from the TCGA database.

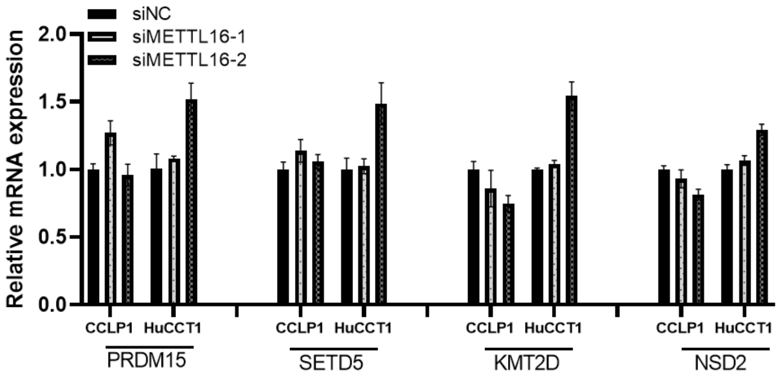

Supplementary Figure 2 (related to Figure. 4) RT-qPCR analysis of PRDM15, NSD2, KMT2D and SETD5 expression in control and METTL16 knockdown cells.

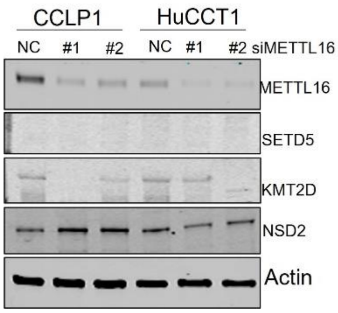

Supplementary Figure 3 (related to Figure. 4). SETD5, KMT2D, and NSD2 protein expression were determined by an immunoblotting assay in control and METTL16 knockdown cells.

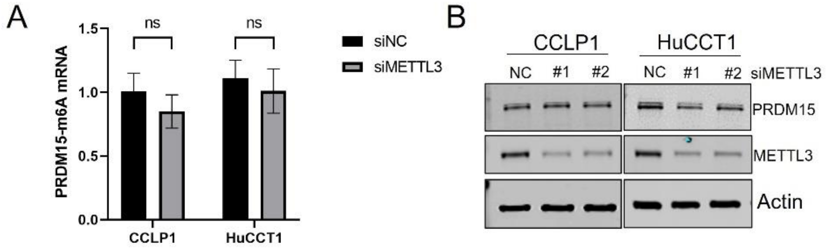

Supplementary Figure 4 (related to Figure. 4). A. MeRIP-qPCR assay was performed in METTL3 silencing (siMETTL3 #1) and control CCLP1 and HuCCT1 cells to detect the level of m6A-modified PRDM15 mRNA. B. PRDM15 protein expression was determined by an immunoblotting assay in control and METTL3 knockdown cells.

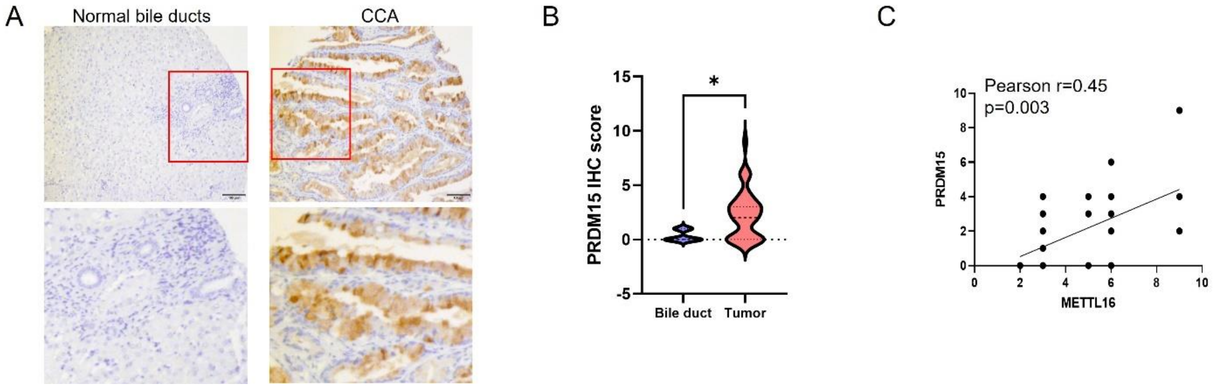

Supplementary Figure 5 (related to Figure. 5) . The expression of PRDM15 and METTL16 is positively correlated in human CCA tissues. (A). Representative immunochemistry (IHC) staining for PRDM15 in normal bile duct and CCA tissues. Scale bar: 100 µm. (B). Quantitative result of the IHC data as represented in A. (C). Positive correlation between PRDM15 and METTL16 expression in CCA patient samples (Pearson correlation coefficient analysis based on the IHC scores).

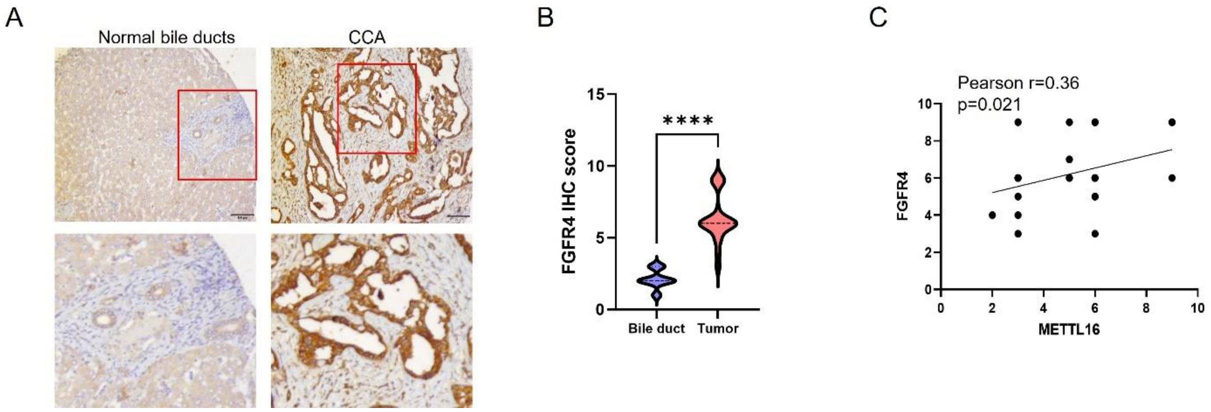

Supplementary Figure 6 (related to Figure. 6) The expression of FGFR4 is positively correlated with METTL16 in CCA tissues. (A). Representative immunochemistry (IHC) staining for FGFR4 expression in human CCA tissues and non-tumorous bile duct. Scale bar: 100 µm. (B). Quantitative result of the IHC data as represented in A. (C). Positive correlation between FGFR4 and METTL16 expression in CCA) patient samples (Pearson correlation coefficient analysis based on the IHC scores).

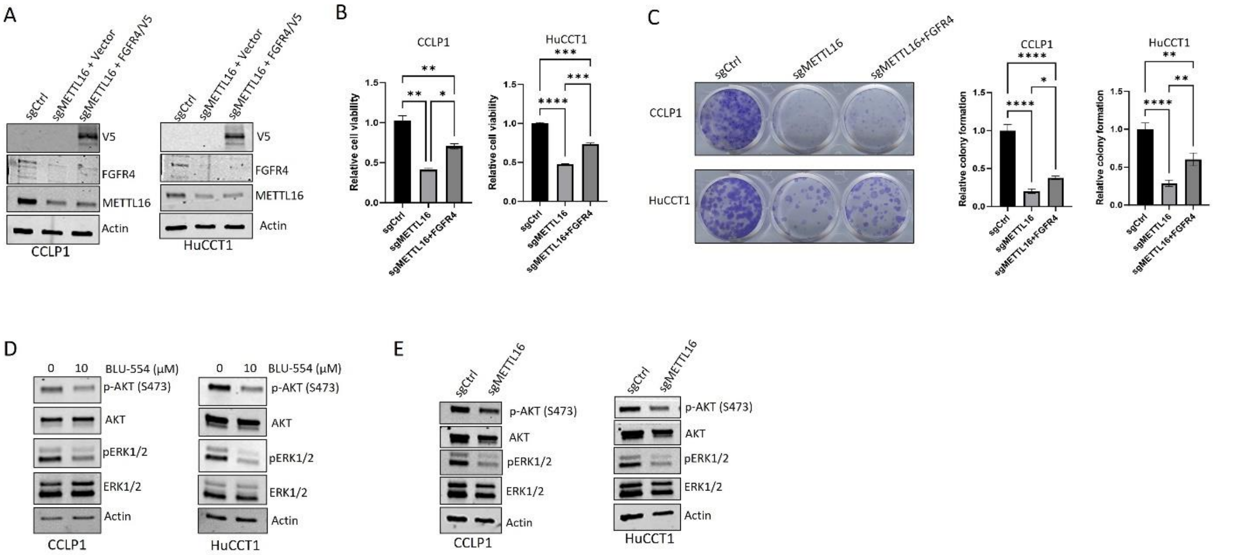

Supplementary Figure 7 (related to Figure. 6) Forced overexpression of FGFR4 rescues the deficiency of CCA cell proliferation and colony formation induced by METTL16 depletion. V5-tagged FGFR4 expression construct was transfected in CCLP1 and HuCCT1 cells for 48 hours and FGFR4 protein expression was determined by immunoblotting (A). WST-1 cell proliferation (B) and colony formation (C) assay were performed in CCLP1 and HuCCT1 cells transfected with FGFR4 overexpression or control construct. (D, E) Phosphorylation of FGFR4 signaling pathway components ERK1/2 and AKT were determined in BLU-554

treated (D) or METTL16 depletion (E) CCA cells by western blotting assay. ***P<0.01*, ****P<0.001*, *****P<0.0001*. (B, C, E, F) Mean ± SD, One-way ANOVA test.
